# Downregulation of *CYP17A1* by 20-hydroxyecdysone: plasma progesterone and its vasodilatory properties

**DOI:** 10.2144/fsoa-2022-0006

**Published:** 2022-07-07

**Authors:** Maneera Y Aljaber, Nelson N Orie, Asmaa Raees, Suhail Kraiem, Mashael Al-Jaber, Waseem Samsam, Mostafa M Hamza, David Abraham, Norman M Kneteman, Alka Beotra, Vidya Mohamed-Ali, Mohammed Almaadheed

**Affiliations:** 1 Anti-Doping Laboratory Qatar, Sports City Road, Doha, 27775, Qatar; 2 Centre of Metabolism & Inflammation, Division of Medicine, Royal Free Campus, University College London, Rowland Hill Street, London, NW3 2PF, UK; 3 Qatar Computing Research Institute, Hamad bin Khalifa University, Doha, 5825, Qatar; 4 KMT Hepatech Inc., PhoenixBio Group, 11421 Saskatchewan Drive, Edmonton, AB, T6G 2M9, Canada; 5 Division of Transplantation Surgery, University of Alberta, Edmonton, Canada

**Keywords:** 20-hydroxyecdysone, *CYP17A1*, ecdysteroid, hepatic transcriptome, humanized liver, mesenteric arteriole, muscle arteriole, progesterone, vasodilation, voltage-dependent calcium entry

## Abstract

**Aim:**

To investigate the effect of 20-hydroxyecdysone on steroidogenic pathway genes and plasma progesterone, and its potential impact on vascular functions.

**Methods:**

Chimeric mice with humanized liver were treated with 20-hydroxyecdysone for 3 days, and hepatic steroidogenic pathway genes and plasma progesterone were measured by transcriptomics and GC–MS/MS, respectively. Direct effects on muscle and mesenteric arterioles were assessed by myography.

**Results:**

*CYP17A1* was downregulated in 20-hydroxyecdysone-treated mice compared with untreated group (p = 0.04), with an insignificant increase in plasma progesterone. Progesterone caused vasorelaxation which was blocked by 60 mM KCl, but unaffected by nitric oxide synthase inhibition.

**Conclusion:**

In the short term, 20-hydroxyecdysone mediates *CYP17A1* downregulation without a significant increase in plasma progesterone, which has a vasodilatory effect involving inhibition of voltage-dependent calcium channels, and the potential to enhance 20-hydroxyecdysone vasorelaxation.

## Plain language summary

The study looked at how the level of the hormone progesterone might be changed by taking a steroid called 20-hydroxyecdysone, which can be found in dietary supplements, and how this might affect the arteries that carry blood to the muscles. The substance was given to mice for 3 days before samples of blood and liver were taken and measured. The progesterone level did not increase appreciably during this short period, but the gene (*CYP17A1*) which produces the enzyme that breaks it down was reduced substantially. This was both able to cause the arteries to expand and to help 20-hydroxyecdysone expand them.

## Graphical abstract

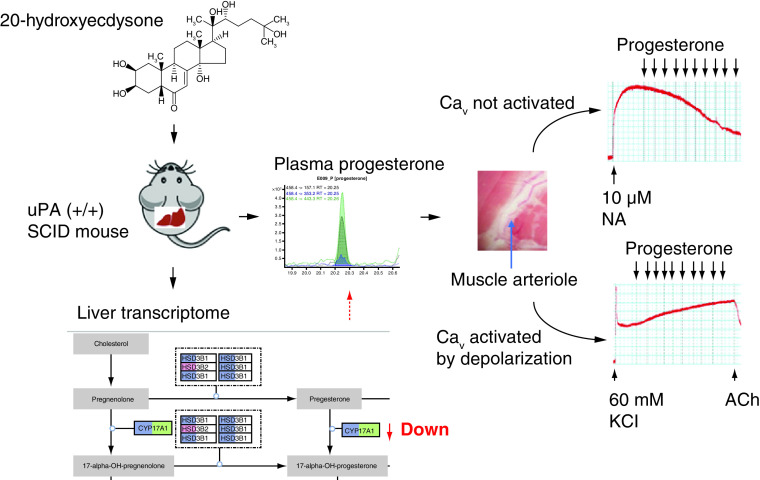

Administration of anabolic–androgenic steroids (AAS) disturbs the regular endogenous production of testosterone and gonadotrophins, with these effects persisting for months after drug withdrawal [1]. In animal studies, administration of testosterone has been shown to suppress the secretion of luteinizing hormone [2] by inhibiting the release of gonadotropin-releasing hormone [3]. Also, administration of high doses of AAS depleted the numbers of cells that produce testosterone (Leydig cells) irreversibly, suggesting a long-lasting alteration [2]. The use of these AAS, with established sport performance-enhancing capabilities, is prohibited in sporting competitions [4]. This has led to the search for potential legal substitutes with similar properties.

Ecdysteroids were originally described in insects as molting hormones but are abundant in plants as phytoecdysteroids. Their most reported biological property is the ability to increase muscle mass, and therefore the potential to enhance physical performance [5–7]. This has made them attractive to elite athletes, with prevalence of their use in this cohort estimated at 0.4–5% [8,9]. In addition to their performance-enhancing properties, ecdysteroids have also been reported to have hormone-disruptive capability [10]. Evidence that supports this includes the similarity in their chemical structures to that of estradiol, which may allow them to bind to estrogen receptors [11]. Also, chronic consumption of supplements containing ecdysteroids appears to alter various steroid hormones, including progesterone, estrogen and dehydroepiandrosterone, in both men and women [10].

An increase in plasma progesterone level can lead to decreased blood pressure [12,13] and blunted pressor response to angiotensin II [14]. These suggest that an increased progesterone level could markedly impact blood vessel functions. Very little is known about how it might impact muscle arterioles, which could have implications for muscle blood flow and performance.

As the concern surrounding their use grows, the World Anti-Doping Agency has now included ecdysteroids, as potential sport dopants, in its monitoring program [15].

Of these compounds, 20-hydroxyecdysone is the most abundant and the most studied. Thus the aims of this study were: to determine whether 20-hydroxyecdysone ingestion induces detectable changes in the mRNA expression of the components of the steroidogenic pathway; if so, whether this leads to increases in plasma progesterone levels; and to record the direct effect of progesterone on muscle and mesenteric arteriolar reactivity.

## Materials & methods

### Reagents & chemicals

Progesterone, 20-hydroxyecdysone (20-OHE), Nω-nitro-l-arginine methyl ester hydrochloride, terbutylmethylether (TBME), NaHCO_3_, K_2_CO_3_, dithioerytritol (DTE) and KCl were purchased from Sigma (Darmstadt, Germany). Human/mouse drug Metabolism Phase I Enzymes, human Drug Metabolism PCR Array and SYBR^®^ Green master mix were purchased from Qiagen (Hilden, Germany). MSTFA and β-glucuronidase from *E. coli* were from Roche (Mannheim, Germany), while orthophosphoric acid was from Merck (Darmstadt, Germany).

### *In vivo* animal studies

Male chimeric uPA(+/+)-severe combined immunodeficiency mice, transplanted with primary human hepatocytes at KMT Hepatech, Inc. (Edmonton, Canada) as previously described [16], were used for experiments. All *in vivo* experiments with these animals were carried out at KMT Hepatech, Inc., as per approval of the National Laboratory Ethics Committee (ECD06/09). Detection of elevated levels of human albumin in the plasma of these mice indicated successful human hepatocyte transplantation or humanization of the liver. Only mice with elevated levels of the human albumin were used for experiments. The mice were then given either 20-OHE (0.2 mg; n = 4) or vehicle (5% ethanol/phosphate-buffered saline; n = 4) by gavage on days 0, 1 and 2 of the experiment, as previously described [8]. The animals were euthanized 2 days after the last treatment, and their livers collected and placed in liquid nitrogen until being shipped to the lab in Qatar for analysis.

### Hepatic gene expression analysis

#### Total RNA extraction

TRIzol™ reagent (Invitrogen, Paisley, UK) was added to finely ground liver tissues to disrupt the cells and release RNA. Isopropanol was added to precipitate the RNA, which was then washed with 70% ethanol. The quality of the RNA was checked using both NanoDrop™ (Thermo Fisher Scientific, DE, USA) and Bioanalyzer (Agilent, CA, USA), and those with RNA integrity number >5.0 were converted to cDNA (using Qiagen first strand cDNA synthesis kit) and the chimeric phenotype confirmed by real-time PCR expression of human β-actin.

#### Hepatic transcriptome analysis

The hepatic transcriptome was analyzed using GeneChip™ WT PLUS Reagent Kit (Thermo Fisher Scientific, Horsham, UK). The first and second strands of cDNA were synthesized from 100 ng of RNA. This was followed by the synthesis and amplification of cRNA overnight. The cRNA was then purified using magnetic beads and the amount determined using the NanoDrop. Next, 15 μg of the purified cRNA was used for the synthesis of second-cycle single-stranded cDNA followed by RNA hydrolysis and removal by RNase H. The single-stranded cDNA was purified, and 5 μg was used as input for the fragmentation and the terminal labeling reactions. In the final step, the labeled fragments were hybridized to the human transcriptome array (Clariom™ S Assay HT; Thermo Fisher Scientific) on the GeneTitan™ machine (Thermo Fisher Scientific) as per manufacturer instructions.

### Measurement of plasma progesterone

#### Sample preparation

##### Plasma

First, 0.6 ml of 10% orthophosphoric acid was added to 0.5 ml of plasma and left on the bench for 10 min. Thereafter, the mixture was ultracentrifuged at 14,674 × g for 20 min and the supernatant taken. Then 0.9 ml of NaHCO_3_/K_2_CO_3_ (w/w:1/2, pH 9–10) and 2 ml of TBME were added to the supernatant and placed on a rolling platform for 20 min. The organic layer was transferred and evaporated to dryness under nitrogen. Finally, 50 μl MSTFA/DTE/NH_4_I (1000:2:1 [v/w/w]) was added to the dried residues and the mixture incubated for 20 min at 65°C.

##### Urine

First, 0.1 ml of 0.1-M phosphate buffer (pH 7) and 25 μl of β-glucuronidase (from *E. coli*) were added to 0.5 ml of urine and incubated for 1 h at 55°C. Thereafter, 0.3 ml of NaHCO_3_/K_2_CO_3_(w/w: 1:2, pH 9–10) and 2 ml of TBME were added and the mixture placed on a rolling platform for 20 min. The organic layer was transferred and evaporated to dryness under nitrogen. Finally, 50 μl MSTFA/DTE/NH4I (1000:2:1 [v/w/w]) was added to the dried residues and the mixture incubated for 20 min at 65°C.

#### Instrumentation

Agilent GC 7890A coupled with Agilent 7010B triple quadrupole MS were used for the analysis. These were equipped with 7693A auto sampler, 10-μl syringe, split/splitless injector (maintained at 280°C) and an SGE™ BPX5 column (TARAJAN Scientific and Medical, Victoria, Australia). The oven temperature program consisted of the following temperatures: 160°C for 0 min, ramped at 10°C/min up to 200°C, then ramped at 2°C/min to 220°C, ramped at 6°C/min to 292°C, 50°C/min up to 310°C and held for 2 min. Injection volume was 1 μl in a split ratio of 1:10. The carrier gas helium flow was 1.1 ml/min, while the MS system was a triple quadrupole system with electron ionization at 70 eV and multiple reaction monitoring acquisition.

### *In vitro* vascular studies

#### Arteriolar preparation

Muscle and mesenteric arterioles (normalized diameters 307 ± 12 and 298 ± 10 μm, respectively) were isolated from the abdominal muscles and mesentery of male sheep euthanized at a government-licensed abattoir in Doha, and therefore no further ethics approval was needed for tissue collection. The sheep were from stock farmed for meat and had food and water provided *ad libitum*. Tissues were collected immediately following euthanasia and were taken to the laboratory in normal physiological salt solution of the following composition: NaCl (112 mM), KCl (5 mM), CaCl_2_ (1.8 mM), MgCl_2_ (1 mM), NaHCO_3_ (25 mM), KH_2_PO_3_ (0.5 mM), NaH_2_PO_3_ (0.5 mM) and glucose (10 mM) and gassed with 95% O_2_/5% CO_2_ to pH 7.4. Segments (2 mm long) of the isolated arterioles were mounted in isometric myographs (510A; Danish Myo Technology A/S, Hinnerup, Denmark) and were pre-tensioned to an equivalent of 100 mm Hg using an automatic normalization protocol, which also determined the vessels’ luminal diameter as previously described [17,18]. The mounted segments were continuously aerated at 37°C and allowed an equilibration period of at least 1 h before experiments. During the equilibration period the segments were contracted with 90 mM KCl and 10–100 μM noradrenaline to test for viability and optimize tissue responsiveness.

#### Assessment of vascular response to progesterone

Following the equilibration period, the arterioles were precontracted with 10 μM noradrenaline to build a stable tone. Thereafter, progesterone (10^-10^ to 10^-5^ M) was added cumulatively, starting with the lowest concentration, with subsequent doses added after the effect of the previous dose had stabilized. To determine the role of nitric oxide in the observed effects, experiments were repeated in the presence of the NOS inhibitor Nω-nitro-l-arginine methyl ester (100 μM). To determine how much of the effect was dependent on blocking voltage-dependent Ca^2+^ entry into the arteriolar smooth muscle, experiments were repeated in 60 mM KCl contracted segments. Data were acquired using Power Lab system and LabChart software (DMT – ADInstruments, Oxford, UK).

### Statistical analysis

Myograph data were analyzed with GraphPad Prism (v. 6.02; GraphPad, CA, USA) and reported as mean ± standard error of the mean. Concentration–response curves were generated on log scale and fitted with the Prism software. The normality of the data was confirmed using the following four tests: Anderson–Darling test, D’Agostino and Pearson test, Shapiro–Wilk test and Kolmogorov–Smirnov test. Student’s unpaired t-test or two-way analysis of variance (with Bonferroni test) were applied as appropriate to test for significant effects or differences. Significance was defined as p < 0.05, and n = number of samples or experiments.

Transcriptome data were analyzed using TAC 4.0 software (ThermoFisher, MA, USA) comparing the 20-OHE-treated group with the vehicle-treated group. Expression analysis settings used were gene-level fold change <-2 or >2, p < 0.05 (analysis of variance method).

## Results

### Minimal effect of short-term 20-hydroxyecdysone treatment on plasma progesterone concentration in the uPA(+l+)-severe combined immunodeficiency mice

**Figure 1.**
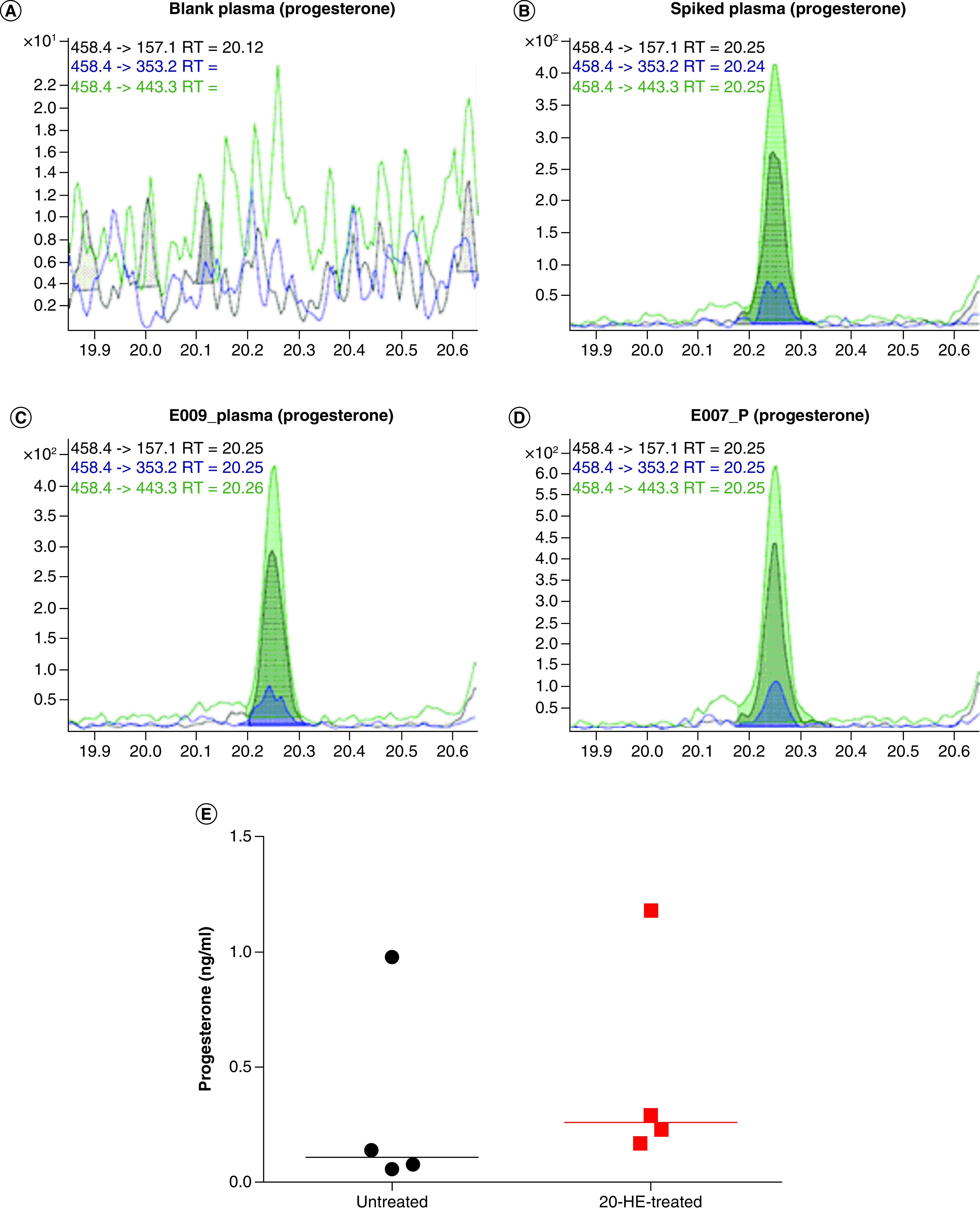
Ion chromatograms (GC–MS/MS, multiple reaction monitoring) for progesterone detection in chimeric severe combined immunodeficiency mice plasma, with and without 20-hydroxyecdysone treatment. **(A)** Blank plasma exempt of progesterone. **(B)** Blank plasma spiked with progesterone (1 ng/ml). **(C)** Plasma from untreated chimeric mouse. **(D)** Plasma from 20-OHE-treated chimeric mouse. **(E)** Summary of the plasma progesterone concentrations in the untreated and 20-OHE treated chimeric mice. 20-OHE: 20-Hydroxyecdysone; RT: Retention time.

The mean plasma progesterone concentrations in the untreated and 20-hydroxyecdysone (20-OHE)-treated mice were 0.31 ± 0.16 and 0.47 ± 0.23 ng/ml, respectively. Although the progesterone concentration was slightly higher in the treated group, the difference was not statistically significant (Figure 1).

Progesterone was not detected in any of the urine samples collected from the same mice.

### Changes in hepatic gene expression following 20-OHE treatment

**Figure 2.**
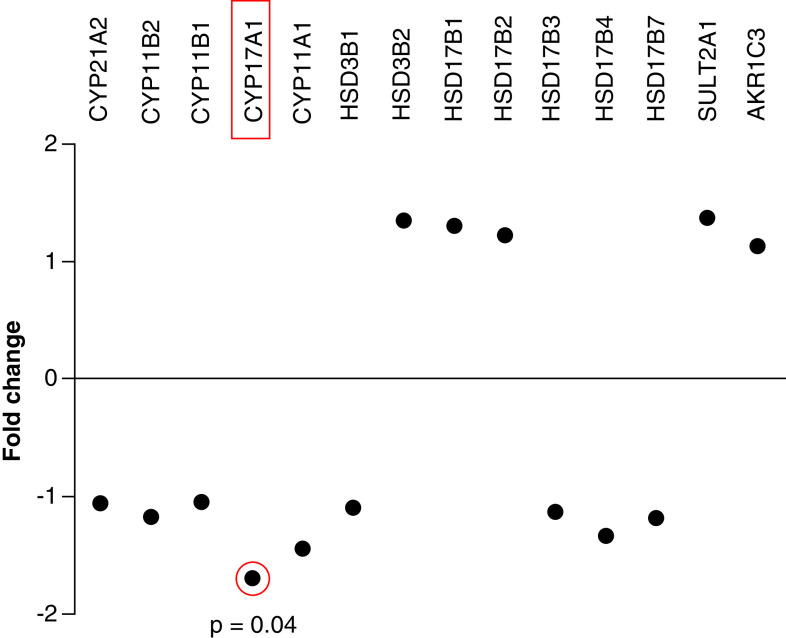
Fold change regulation of steroidogenesis pathway genes in the humanized liver of 20-hydroxyecdysone-treated chimeric mice. *CYP17A1* (highlighted) was significantly downregulated (p = 0.04) in 20-hydroxyecdysone-treated mice compared with untreated controls.

**Figure 3.**
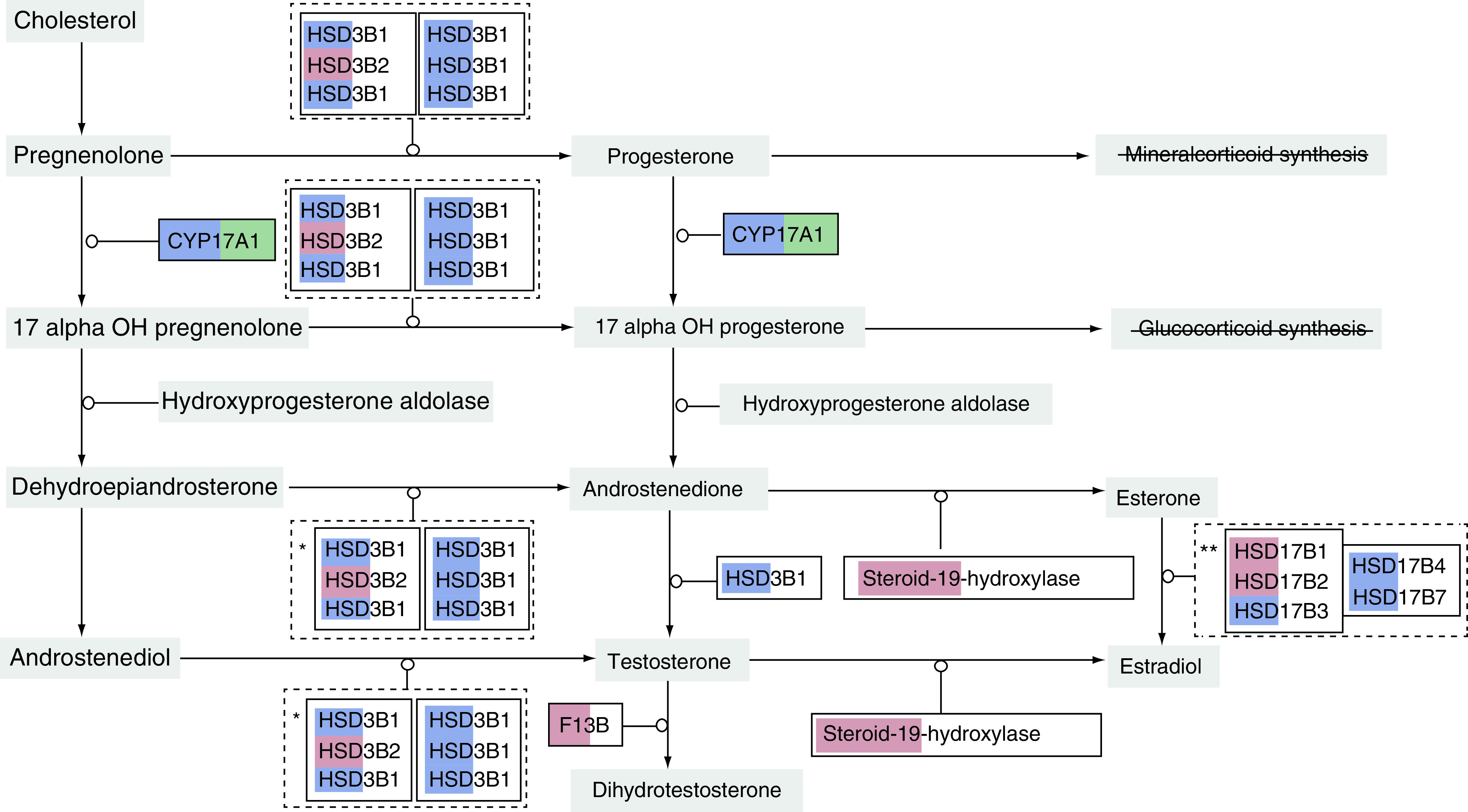
Expression of steroidogenesis pathway genes in the humanized liver of 20-hydroxyecdysone-treated chimeric mice. *CYP17A1* (highlighted in green) was significantly downregulated. The red and blue colors highlight over- and under-representation, respectively, of the genes in the treatment group compared with the untreated control. Pathway analysis was conducted using TAC 4.0 software.

The liver steroidogenesis pathway genes were altered by 20-OHE treatment. Five genes were overexpressed and nine underexpressed in the 20-OHE-treated mice compared with the untreated control group (Figure 2). Notably, *CYP17A1* (p = 0.04) was downregulated (Figures 2 & 3).

### Vasorelaxation induced by progesterone

**Figure 4.**
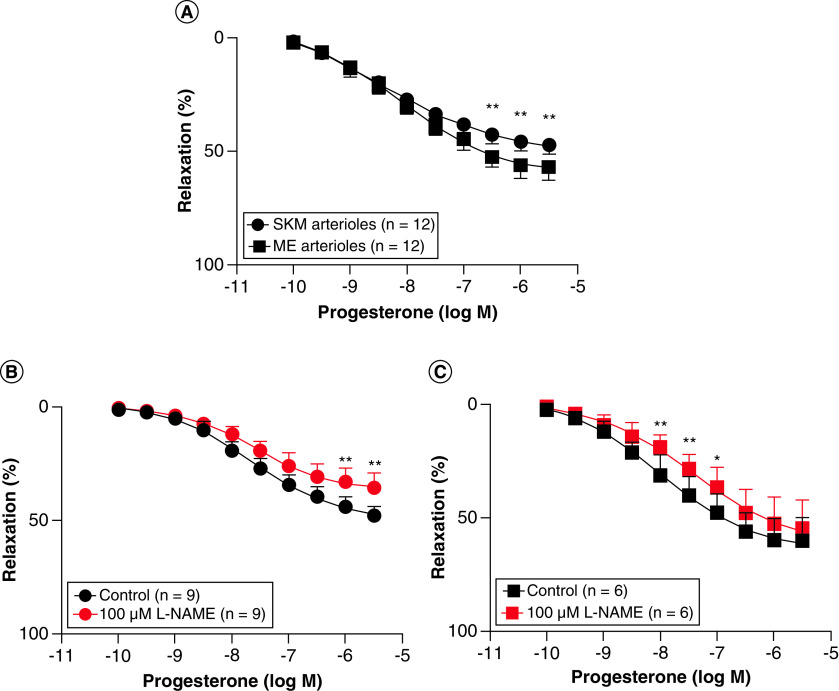
Progesterone-induced vasorelaxation in muscle and mesenteric arterioles. **(A)** Progesterone caused concentration-dependent relaxation in both muscle and mesenteric arterioles. Although the two curves were statistically comparable, maximum relaxation was greater in mesenteric compared with muscle arterioles. **(B)** Effect of NOS inhibition with 100 μM L-NAME in muscle arterioles. **(C)** Effect of nitric oxide synthase inhibition with 100 μM L-NAME in mesenteric arterioles. *p < 0.05; **p < 0.01 compared with control response. L-NAME: Nω-nitro-L-arginine methyl ester; ME: Mesenteric; SKM: Skeletal muscle.

Comparable vasorelaxation was recorded for progesterone in both muscle and mesenteric arterioles (Figure 4A). The maximum relaxation recorded was 44.68 ± 5.07% in muscle (n = 9) and 51.86 ± 7.24% (n = 9) in mesenteric arterioles obtained from the same animals. The difference in maximum relaxation was not statistically significant. The sensitivities (log IC_50_) of both arterioles to progesterone were also similar (Table 1).

**Table 1.** Maximum relaxation and sensitivity (log IC_50_) of muscle and mesenteric arterioles to progesterone.

	Progesterone	Progesterone	p-value
	Muscle arteriole (n = 9)	Mesenteric arteriole (n = 9)	
R_max_ (%)	44.68 ± 5.07	51.86 ± 7.24	ns
Log IC_50_	-7.86 ± 0.22	-8.14 ± 0.24	ns

p-value = compared with muscle arteriole.

ns: Not significant; R_max_: Maximum relaxation.

### Minimal role for nitric oxide in progesterone vasorelaxation

In the presence of the NOS inhibitor Nω-nitro-l-arginine methyl ester, the relaxation curves for progesterone were slightly but insignificantly shifted to the right in both arterioles (Figure 4B & C). However, under these conditions, maximum relaxation to progesterone was significantly reduced in the muscle arterioles (p < 0.01; n = 9; Table 2). The sensitivities (log IC_50_) of both arterioles to progesterone were unchanged by Nω-nitro-l-arginine methyl ester (Table 2).

**Table 2.** Maximum relaxation and sensitivity (log IC_50_) to progesterone in the absence and presence of 100 μM Nω-nitro-l-arginine methyl ester in muscle and mesenteric arterioles.

	Muscle arteriole (n = 9)	+100 μM L-NAME (n = 9)	p-value	Mesenteric arteriole (n = 6)	+100 μM L-NAME (n = 6)	p-value
R_max_ (%)	48.06 ± 4.10	35.67 ± 6.66	0.01	60.10 ± 10.00	54.64 ± 12.10	ns
Log IC_50_	-7.48 ± 0.26	-7.50 ± 0.21	ns	-7.9 ± 0.17	-7.32 ± 0.37	ns

p-value = compared with respective control response in the same arteriole.

L-NAME: Nω-nitro-l-arginine methyl ester; ns: Not significant; R_max_: Maximum relaxation

### Dependence of progesterone-induced relaxation on blocking of voltage-dependent Ca^2+^ entry into the smooth muscle of muscle & mesenteric arterioles

**Figure 5.**
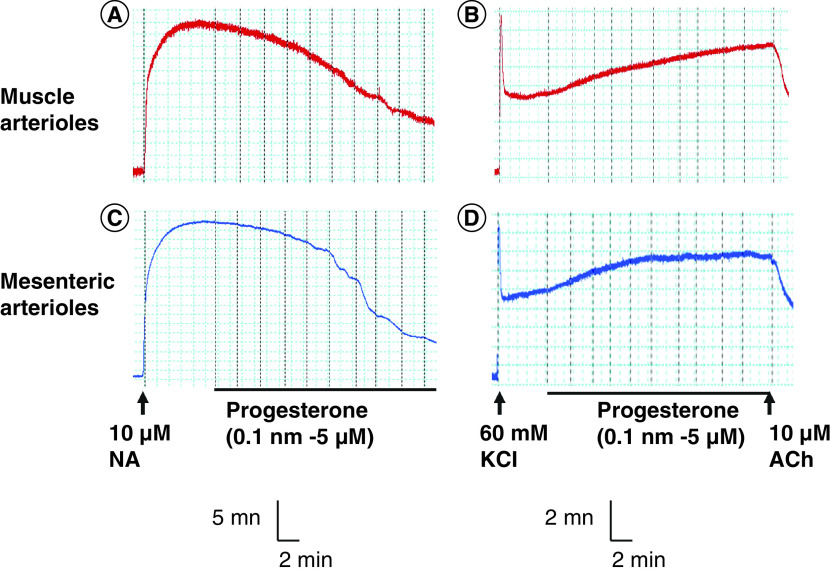
Dependence of progesterone-induced vasorelaxation on contractile agonist. **(A)** Typical concentration-dependent relaxation to progesterone in muscle arteriole contracted with 10 μM NA. **(B)** The absence of relaxation to progesterone in the range applied (up to 5 μM) in muscle arteriole contracted with 60 mM KCl. ACh (10 μM) relaxation was still recorded under this condition. **(C)** Typical concentration-dependent relaxation to progesterone in mesenteric arteriole contracted with 10 μM NA. **(D)** The absence of relaxation to progesterone in the range applied (up to 5 μM) in mesenteric arteriole contracted with 60 mM KCl. ACh (10 μM) relaxation was still recorded under this condition. ACh: Acetylcholine; NA: Noradrenaline.

Given that nitric oxide appeared not to contribute significantly to progesterone relaxation in these arterioles, we examined whether the compound was acting by directly interfering with voltage-dependent Ca^2+^ entry into the arteriolar smooth muscle cells. To do this, the arterioles were exposed to 60 mM KCl in the place of noradrenaline, to depolarize their smooth muscle membrane and contract them by opening the voltage-dependent Ca^2+^ channels [19]. This treatment abolished the relaxation of both arterioles by progesterone. Figure 5A–D shows typical recordings of progesterone-induced relaxation of arteriolar segments contracted by 10 μM noradrenaline versus 60 mM KCl.

### Effect of progesterone on 20-OHE vasorelaxation

**Figure 6.**
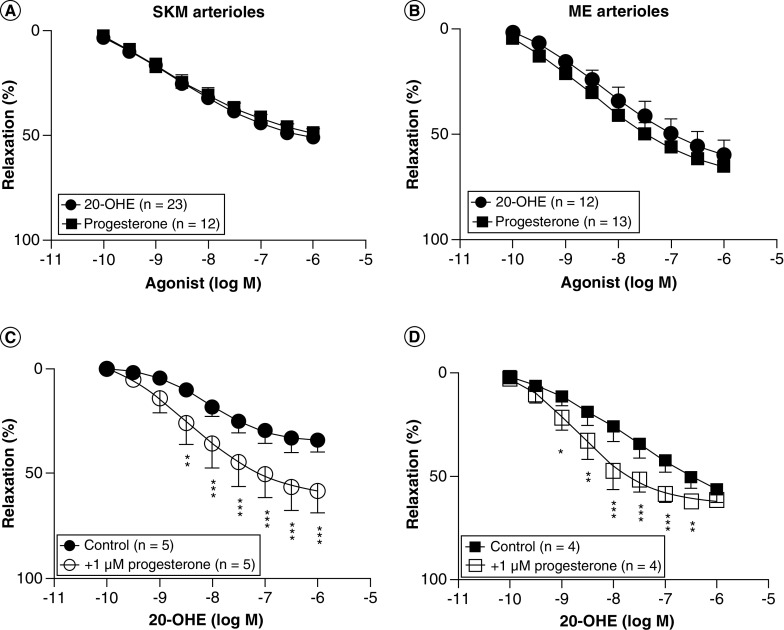
Effect of progesterone on 20-hydroxyecdysone-induced relaxation. **(A)** Comparable relaxation produced by 20-OHE and progesterone when applied separately to muscle arterioles. **(B)** Comparable relaxation produced by 20-OHE and progesterone when applied separately to mesenteric arterioles. **(C)** Enhanced relaxation to 20-OHE in the presence of 1 μM progesterone in muscle arterioles. **(D)** Enhanced relaxation to 20-OHE in the presence of 1 μM progesterone in mesenteric arterioles. *p < 0.05; **p < 0.01; ***p < 0.001 compared with control response. 20-OHE: 20-hydroxyecdysone; ME: Mesenteric; SKM: Skeletal muscle.

When applied separately, 20-OHE and progesterone caused comparable relaxation of muscle (Figure 6A) and mesenteric (Figure 6B) arterioles. Maximum relaxation and sensitivity to both compounds are shown in Table 3.

**Table 3.** Maximum relaxation and sensitivity (log IC_50_) of muscle and mesenteric arterioles to progesterone compared with 20-hydroxyecdysone.

	Progesterone	20-OHE	p-value	Progesterone	20-OHE	p-value
	Muscle arteriole (n = 23)	Muscle arteriole (n = 12)		Muscle arteriole (n = 23)	Muscle arteriole (n = 12)	
R_max_ (%)	50.17 ± 3.71	50.55 ± 3.92	ns	66.94 ± 5.63	59.77 ± 7.03	ns
Log IC_50_	-8.29 ± 0.10	-8.33 ± 0.20	ns	-8.32 ± 0.24	-8.16 ± 0.20	ns

p-value = compared with progesterone in the same arteriole.

20-OHE: 20-hydroxyecdysone; ns: Not significant; R_max_: Maximum relaxation.

In separate experiments where arterioles were primed with progesterone (1 μM), the relaxation to 20-OHE was enhanced in both muscle (Figure 6C) and mesenteric (Figure 6D) arterioles. However, maximum relaxation was only enhanced in the muscle arterioles (Table 4).

**Table 4.** Maximum relaxation and sensitivity (log IC_50_) to 20-hydroxyecdysone in the absence and presence of 1 μM progesterone in muscle and mesenteric arterioles.

	Muscle arteriole (n = 5)	+1 μM progesterone (n = 5)	p-value	Mesenteric arteriole	+1 μM progesterone	p-value
R_max_ (%)	34.26 ± 5.46	58.51 ± 10.21	0.0001	52.03 ± 6.57	55.00 ± 6.86	ns
Log IC_50_	-7.97 ± 0.16	-8.27 ± 0.23	ns	-8.01 ± 0.20	-8.70 ± 0.25	0.01

p-value = compared with respective control response in the same arteriole.

20-OHE: 20-hydroxyecdysone; ns: Not significant; R_max_: Maximum relaxation

## Discussion

The potential for 20-hydroxyecdysone (20-OHE) to cause hormone disruption was studied in chimeric mice with humanized liver, which were treated with 20-OHE for only 3 days and euthanized on day 4 due to ethical considerations related to their immune-compromised state. Transcriptional analysis of the liver revealed the downregulation of *CYP17A1*, which encodes an enzyme involved in the metabolism of progesterone, in the group treated with 20-OHE compared with untreated controls. However, only a small (statistically insignificant) increase in plasma progesterone was associated with this change within the short experimental period. Given that chronic consumption of supplements containing ecdysteroid has been associated with steroid hormone disruption, including increased plasma progesterone [10], the current experimental period was probably too short for a noticeable change in plasma progesterone to occur.

*CYP17A1* catalyzes two key reactions: one is associated with 17-α-hydroxylase activity, converting pregnenolone to 17-hydroxypregnenolone and progesterone to 17-hydroxyprogesterone [20], while the other is its 17,20-lyase activity, which converts 17-hydroxypregnenolone to dehydroepiandrosterone [10,20]. Thus the downregulation of *CYP17A1* by 20-OHE in the current study suggests a potential to cause the accumulation of progesterone *in vivo*, which could increase substantially over time. This would be consistent with the tendency for chronic consumption of supplements containing ecdysteroid to cause steroid hormone disruption, including increased plasma progesterone [18], although the mechanism has not been identified. Previous data have also shown an increased level of estrogen and a decreased level of dehydroepiandrosterone in both men and women taking supplements containing ecdysteroids [10]. The current data have for the first time identified the potential molecular mechanism that would explain increased progesterone levels in consumers of supplements containing 20-OHE.

The other question we sought to answer was what effect any rise in progesterone would have on muscle arteriolar functions and whether this would alter the vasodilatory effects of 20-OHE [21]. The data showed that progesterone was able to dilate both muscle and mesenteric arterioles, as well as enhancing the vasodilatory effect of 20-OHE in these arterioles. The focus on the muscle arterioles was because of their relevance to sports performance. The test of an additional vessel type (mesenteric) was to determine whether the effect was vascular bed-specific or more generalized. The data also showed that these effects were more dependent on directly blocking voltage-dependent Ca^2+^ entry into the vascular smooth muscle cells, rather than the release of nitric oxide in both vessel types.

The Ca^2+^ required for vascular smooth muscle contraction is mobilized from the extracellular space and/or released from the sarcoplasmic reticulum depending on the stimulus. K^+^-induced contraction occurs by the depolarization of the vascular smooth muscle membrane, which activates Ca^2+^ entry mainly through voltage-dependent (L-type) calcium channels [22]. On the other hand, noradrenaline-induced contraction occurs by both the mobilization of Ca^2+^ from the extracellular space via receptor-operated membrane channels, and release from the sarcoplasmic reticulum [19,23,24]. While progesterone could relax both muscle and mesenteric arterioles precontracted with 10 μM noradrenaline, it failed to relax the same arterioles when they were contracted with 60 mM KCl. This observation suggests that the high K^+^ condition was hindering the ability of progesterone to reduce the cytosolic Ca^2+^ concentration, as required to cause relaxation of these arterioles. Because high K^+^ causes contraction mainly by promoting Ca^2+^ entry through L-type channels [22], the failure of progesterone to cause relaxation under this condition would be consistent with a compound that selectively blocks these channels in the vascular smooth muscle [25]. Although the blocking of voltage-dependent, nonselective cation channels cannot be ruled out, the data are consistent with the mechanism of progesterone relaxation of these arterioles being, in large part, dependent on the blocking of Ca^2+^ entry through the voltage-dependent (L-type) calcium channels in the vascular smooth muscle of these arterioles.

Although progesterone did not require nitric oxide to relax these arterioles, its presence enhanced the relaxation to 20-OHE, which mechanism is largely nitric oxide-dependent [21]. The synergy may be related to the utilization of different but complementary mechanisms of relaxation by the two compounds in these arterioles. The exact mechanism of this synergy will require further investigation.

## Conclusion

This study has for the first time identified a potential mechanism by which plasma progesterone levels might be increased by the ingestion of 20-hydroxyecdysone found in plants and sports supplements. The study also demonstrated that progesterone can both dilate muscle arterioles and enhance the vasodilatory property of 20-hydroxyecdysone, both of which have the potential to increase muscle blood flow and performance.

**Summary points**

**Introduction**

- The prohibition of the use of anabolic androgenic steroids in competitive sports has led to the search for potential legal substitutes with similar properties. Ecdysteroids, which are abundant in plants as phytoecdysteroids, are one of such substitutes with the ability to increase muscle mass, and therefore attractive to elite athletes. However, chronic consumption of supplements containing ecdysteroids can alter endogenous production of various steroid hormones, including progesterone, in both men and women. The mechanism of this hormone disruption is not known and very little is known about how it might impact muscle blood vessel function.

**Aim**

- (1) To determine whether the ingestion of the most common ecdysteroid, 20-hydroxyecdysone induces detectable changes in the mRNA expression of the components of the steroidogenic pathway. (2) To determine whether such ingestion leads to an increase in plasma progesterone level. (3) To record the direct effect of progesterone on muscle and mesenteric arteriolar reactivity.

**Materials & methods**

- Chimeric uPA+/+-SCID mice with humanized liver were treated with 20-hydroxyecdysone for 3 days. At the end of this period, the mice were euthanized, and blood and liver samples were collected and analyzed. Changes in hepatic steroidogenic pathway genes and plasma progesterone concentrations were measured by transcriptomics and GC-MS/MS, respectively. Direct effects of progesterone on muscle and mesenteric arterioles were assessed by wire myography.

**Results**

- Hepatic steroidogenesis pathway genes were altered by 20-OHE treatment; Notably, CYP17A1 gene was downregulated in 20-hydroxyecdysone-treated compared with untreated group. However only a small (insignificant) increase in plasma progesterone concentration was recorded in the short-term. Application of progesterone to both muscle and mesenteric arterioles caused them to relax in a manner that was completely blocked by exposure to high potassium chloride (60 mM) concentration but was unaffected by nitric oxide synthase inhibition. In addition, pre-exposure of the arterioles to progesterone enhanced their relaxation to 20-hydroxyecdysone.

**Discussion/conclusion**

- This study has for the first time identified CYP17A1 gene downregulation as a potential mechanism by which plasma progesterone levels might be increased by the ingestion of 20-hydroxyecdysone. The study also demonstrates that progesterone can both dilate muscle arterioles as well as enhance the vasodilatory property of 20-hydroxyecdysone, both of which have the potential to increase muscle blood flow and performance. The vasodilatory effect of progesterone in these arterioles involves the blocking of voltage-dependent calcium entry.
